# Age-Dependent Burst Suppression During Anesthesia in Young Children with Congenital Heart Disease: The Impact of Anesthetic Depth

**DOI:** 10.3390/children12101401

**Published:** 2025-10-17

**Authors:** Annelie Augustinsson, Carina Sjöberg, Johan Holmén, Anders Hjärpe, Pether Jildenstål

**Affiliations:** 1 Care in High Technological Environments, Department of Health Sciences, Lund University, SE-221 00 Lund, Sweden; carina.sjoberg@med.lu.se (C.S.); pether.jildenstal@med.lu.se (P.J.); 2 Department of Anesthesiology and Intensive Care, Institute for Clinical Sciences, Sahlgrenska Academy, University of Gothenburg, SE-405 30 Gothenburg, Sweden; 3 Queen Silvia Children’s Hospital, Sahlgrenska University Hospital, SE-416 50 Gothenburg, Sweden; 4 Department of Perfusion, Sahlgrenska University Hospital, SE-416 50 Gothenburg, Sweden; 5 Department of Anesthesiology and Intensive Care, Skåne University Hospital, SE-221 85 Lund, Sweden; 6 Institute of Health and Care Sciences, Sahlgrenska Academy, University of Gothenburg, SE-405 30 Gothenburg, Sweden; 7 Department of Anesthesiology and Intensive Care, School of Medical Science, Örebro University Hospital, SE-701 85 Örebro, Sweden; 8 Faculty of Nursing and Health Sciences, Nord University, NO-8022 Bodø, Norway

**Keywords:** burst suppression, electroencephalography, general anesthesia, pediatric surgery, spectral edge frequency, thoracic surgery

## Abstract

**Highlights:**

**What are the main findings?**

- Higher spectral edge frequency (SEF) was associated with lower burst suppression (BS) throughout the surgical procedure.
- Children under 12 months showed a stronger SEF–BS correlation compared to those aged 12–36 months.

**What is the implication of the main findings?**

- SEF appears to be an age-sensitive indicator of anesthetic depth during sevoflurane anesthesia with extracorporeal circulation.
- These findings underscore the importance of individualized, age-adjusted anesthesia monitoring strategies in pediatric cardiac surgery.

**Abstract:**

**Background/Objectives**: Electroencephalography (EEG) is increasingly used in pediatric anesthesia to detect abnormal brain activity such as burst suppression (BS), a marker of profound cortical inactivation. The objective of this study was to assess anesthetic depth using bilateral spectral edge frequency (SEF) and to determine the incidence of frontal cortical BS in young children undergoing cardiac surgery with extracorporeal circulation (ECC) under sevoflurane anesthesia. **Methods**: Twelve children, divided into two age groups (<12 months and 12–36 months), were included. EEG sensors were placed on the forehead and continuously monitored with SedLine^®^. BS and SEF were analyzed using linear mixed-effects models, accounting for age group and repeated measurements across the procedure. **Results**: BS did not differ significantly over time. Across the full surgical procedure, higher SEF was associated with lower BS. However, children <12 months exhibited a stronger SEF–BS relationship, suggesting greater susceptibility to BS compared to older children. Before and during ECC, SEF and age group were not significantly related to BS. Random effects indicated moderate to substantial between-subject variability. Scatterplots showed a negative SEF–BS relationship overall, but weak and inconsistent associations during specific perioperative phases, underscoring the phase-dependent nature of SEF–BS dynamics. **Conclusions**: SEF is an age-sensitive marker of anesthetic depth during sevoflurane anesthesia with ECC, with children <12 months showing greater susceptibility to BS. These findings highlight the importance of individualized, age-adjusted anesthesia monitoring strategies in pediatric cardiac surgery.

## 1. Introduction

Congenital heart disease (CHD) is the most common birth defect worldwide. In developed countries, advances in diagnostics, surgery, and post-operative care have led to more than 90% of children with CHD surviving into adulthood [1]. Since the early 2000s, survival rates have plateaued at around 97%. Despite this, early mortality remains high for children with complex CHD, while efforts for those with less severe forms focus on preventing acquired complications [2].

In Sweden, data from the Swedish National Registry on Congenital Heart Disease (SWEDCON) show that 750–1000 newborns (approximately 1%) are diagnosed with CHD each year. The most common surgical cases involve shunt defects such as atrial septal defect (ASD) and ventricular septal defect (VSD). In 2023, 466 children underwent cardiac surgery, with heart-lung machines used in 89% of the procedures [3]. A systematic review and meta-analysis found that early surgery in children with complex CHD, especially single-ventricle cases, is associated with an increased risk of impaired developmental outcomes [4]. General anesthesia is essential for managing surgical stress and pain. However, current approaches to individualizing anesthesia depth, relying on indirect markers such as pharmacokinetic models, minimum alveolar concentration (MAC), effect-site concentration, and autonomic signs, e.g., heart rate (HR) and blood pressure, remain insufficiently accurate or reliable [5].

General anesthesia is a drug-induced state of unconsciousness, but there remains uncertainty about how best to safely and reversibly manipulate the central nervous system [6]. It affects hemodynamics by causing hypotension and reducing blood flow, which can compromise oxygen delivery to vital organs [7]. Importantly, traditional indicators such as heart rate and blood pressure do not accurately reflect cortico-thalamic activity or the depth of the hypnotic state [8]. Electroencephalogram (EEG) monitoring provides real-time visualization of brain activity. When performed with SedLine^®^ (Masimo™), it additionally generates the processed Patient State Index (PSi), a calculated measure of brain activity. This allows clinicians to more accurately assess anesthetic depth and reduce the risk of burst suppression (BS) in pediatric patients [9]. EEG-guided anesthesia has been shown to reduce sevoflurane requirements and may decrease the incidence of BS in children aged one to six years classified as ASA Physical Status Class I–II (under the American Society of Anesthesiologists Physical Status Classification System) while undergoing minor surgical procedures [8]. Additionally, elevated sevoflurane concentrations, particularly above 3% during induction, have been associated with EEG discontinuities in infants and young children undergoing elective procedures [10].

EEG-guided anesthesia has been shown to enable continuous personalization and reduce the risk of discontinuity patterns in neonates, which are associated with elevated sevoflurane doses and intraoperative hypothermia [11]. Discontinuous EEG activity in children may reflect either cerebral immaturity or a deeper level of anesthesia. These patterns are age-dependent and evolve with maturation, offering important insights into underlying neurodevelopmental processes [12]. Together, these findings underscore the heightened cerebral vulnerability of children with CHD, especially during prolonged open-heart surgery involving extracorporeal circulation (ECC), where the systemic inflammatory response further increases the risk of adverse neurocognitive outcomes [13,14]. Despite these findings, there remains a lack of research on how to optimize anesthetic depth and prevent BS in pediatric patients with CHD, especially in those undergoing prolonged surgeries or presenting with pre-existing cerebral vulnerabilities.

The objectives of this study were to assess the depth of anesthesia by measuring bilateral spectral edge frequency (SEF) and to determine the incidence of frontal cortical BS in young children undergoing thoracic surgery with ECC under sevoflurane anesthesia.

## 2. Materials and Methods

### 2.1. Study Design

The current study was a single-center, non-invasive observational study. The study was conducted in accordance with the Declaration of Helsinki and Good Clinical Practice (GCP) guidelines, as well as all applicable laws and regulations. Ethical approval was obtained from the Swedish Ethical Review Authority (protocol code 1066-18) on 30 December 2018. Prior to participation, informed consent was obtained from the legal guardians of all patients, as appropriate for the pediatric population involved. The study was prospectively registered on ClinicalTrials.gov (identifier NCT04206683) on 18 December 2019.

### 2.2. Setting and Participants

The study was performed at Queen Silvia Children’s Hospital, Sahlgrenska University Hospital, Gothenburg, Sweden. Twelve children scheduled for surgery for ASD and/or VSD, including patent ductus arteriosus (PDA), (under ASA Class II) under general anesthesia were included in the study between 20 January 2020 and 30 June 2024. Written informed consent was obtained from parents/legal guardians. Inclusion criteria were children aged 6 to 36 months undergoing open-heart surgery with the support of ECC. Exclusion criteria included dark skin pigmentation, neurological conditions such as stroke, epilepsy, brain tumor, brain or eye hemangioma, children at risk of undergoing surgery during on-call hours, frontal skin lesions, and parents who did not understand spoken and written Swedish.

Anesthesia induction was performed in accordance with the clinic’s standard protocol. Sevoflurane inhalation was used in cases where vascular access had not been established beforehand. Otherwise, intravenous induction was carried out using propofol (2–5 mg/kg), ketamine (2–5 mg/kg), or esketamine (1–3 mg/kg) in combination with midazolam (0.1 mg/kg), depending on the patient’s cardiac condition and overall hemodynamic status. All patients received fentanyl (2–3 µg/kg) and atracurium (0.5 mg/kg) prior to endotracheal intubation. Maintenance of anesthesia was based on either sevoflurane or propofol infusion, in combination with repeated doses of fentanyl and atracurium.

### 2.3. Data Collection

All patients were monitored with a sensor for EEG bilateral SEF monitoring (SedLine^®^, Masimo Corporation, Irvine, CA, USA) to identify risk for BS. The sensors were placed on the child’s frontal head, as soon as the situation allowed. Most patients had their sensor connected before or immediately after endotracheal intubation. In some cases, sensors were placed prior to anesthesia induction. After anesthesia induction and tracheal intubation, arterial and central lines were placed and a urinary catheter inserted. Values of EEG/PSi, bilateral SEF, oxygen saturation (SpO_2_), HR, invasive MAP, end-tidal carbon dioxide (ETCO_2_), and temperature (central and peripheral) were registered. To avoid causing stress to the children prior to induction, no baseline values before anesthesia were collected.

Patient monitoring data and ECC variables were registered automatically every 20 s with the LivaNova Connect patient monitoring and data management system (LivaNova). ECC was performed using a Stöckert S5 heart-lung machine equipped with roller pumps and a Stöckert Heater-Cooler System 3T (LivaNova, Mirandola, Italy). ECC circuit components were selected based on the patient’s estimated flow requirements during bypass. The tubing setup consisted of a 1/4″ venous line and either a 3/16″ or 1/4″ arterial line. A dialysis filter (Terumo Corp., Tokyo, Japan) was integrated into all circuits. Oxygenators and hard-shell venous reservoirs that were used included the Capiox FX-05 (Terumo Corporation, Tokyo, Japan) and the Affinity Pixi (Medtronic, Minneapolis, MN, USA). The circuit was initially primed with 300 mL of Ringer’s Acetate and 100 mL of Tribonat^®^ (Fresenius Kabi AB, Uppsala, Sweden). After removing the pre-bypass filter, 100 mL of albumin (200 mg/mL) and 1000–2000 IU of heparin was added to the priming solution. The target hematocrit during ECC was maintained between 24% and 30%. If the calculated hematocrit was below 24%, packed red blood cells were added to achieve a hematocrit of approximately 30%. Following the addition of albumin and red blood cells, pre-bypass filtration was employed to reduce the priming volume to a total of 380–400 mL.

Prior to cannulation, a heparin induction dose was administered via the central venous line. The dose was calculated by the perfusionist based on body weight at 350 IU/kg, rounded to the nearest 500 IU, resulting in a total dose of approximately 350–400 IU/kg. Activated clotting time (ACT) was measured using the Hemochron Signature Elite (Accriva, San Diego, CA, USA). A baseline ACT was obtained from a radial or femoral arterial sample prior to cannulation. An ACT > 400 s was deemed sufficient for cannulation and an ACT > 480 s was required for initiation of cardiopulmonary bypass (CPB). ACT was reassessed 20–30 min after ECC initiation and monitored throughout the procedure using samples drawn from the ECC circuit. All patients underwent aortic root cannulation, followed by bicaval venous cannulation. Cannula sizes were selected according to manufacturer-provided flowcharts, based on calculated pump flow requirements. CPB was initiated and maintained at a target non-pulsatile pump flow rate of 2.8–3.0 L/min/m^2^. Cardiac arrest was achieved using cold blood cardioplegia. An initial induction dose of 20–30 mL/kg body weight was administered, with repeat doses of 10 mL/kg body weight given every 20–30 min as needed. Blood gas parameters during ECC were continuously monitored using the CDI^®^ Blood Parameter Monitoring System 550 (Terumo Cardiovascular Group, Ann Arbor, MI, USA). The target arterial oxygen tension (pO_2_) during CPB was maintained at 20–25 kPa, and arterial carbon dioxide tension (pCO_2_) at 5–6 kPa. Following weaning from ECC, systemic heparinization was reversed using protamine sulfate, administered at a 1:1 ratio relative to the initial heparin dose.

### 2.4. Statistical Analysis

Patients were stratified into two age groups (<12 months and 12–36 months), and time was subcategorized into three surgical phases: before ECC, during ECC, and after ECC. The period “after ECC” was included in the full surgical procedure analyses but was not analyzed as a separate subgroup.

Patient monitoring data and ECC variables were automatically recorded every 20 s. For analysis covering the full surgical procedure, data was down sampled to 10 min intervals by selecting the closest measurement within each interval to reduce dataset size and highlight overall trends. In contrast, for the shorter “before ECC” and “during ECC” periods, all available time points (every 20 s) were retained to preserve the temporal resolution necessary to capture rapid physiological changes.

**Figure 1:**
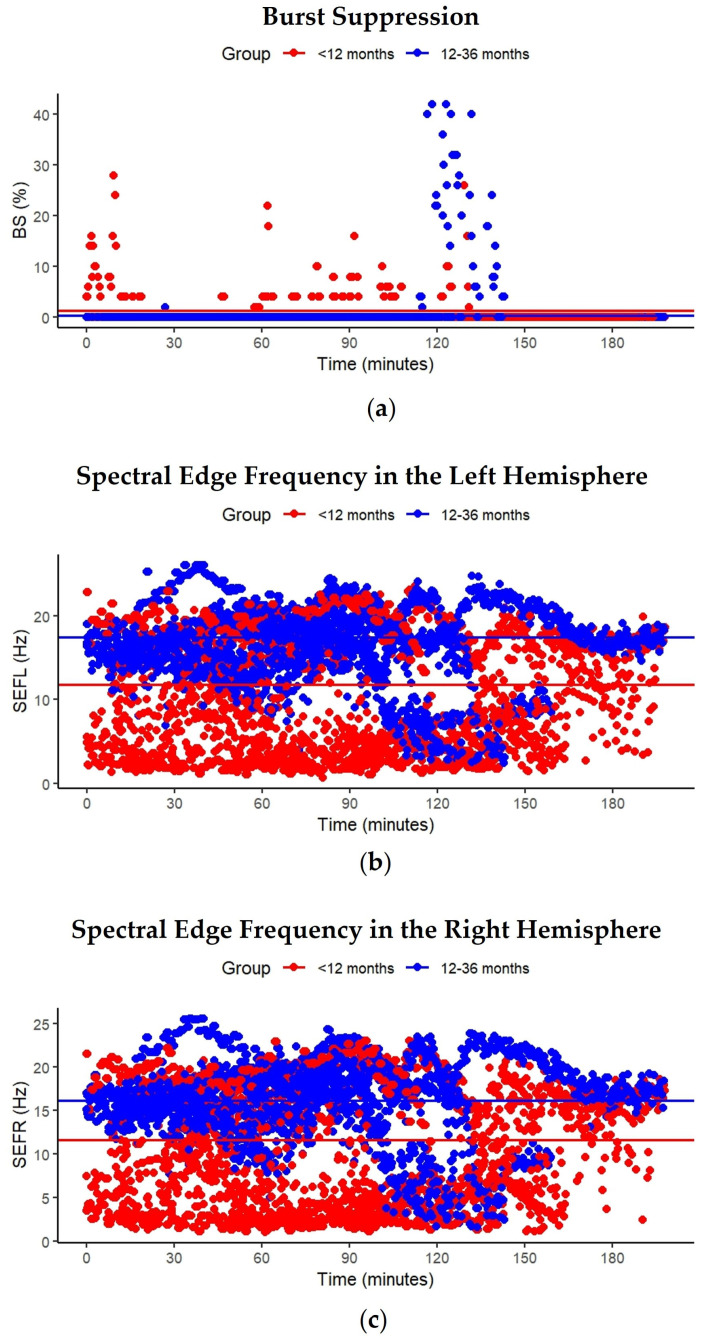
Temporal patterns of burst suppression (BS) and spectral edge frequency (SEF). Scatterplots showing (**a**) BS with an intercept *p*-value (*P*_intercept_) of 0.9; (**b**) SEF from the left hemisphere (SEFL) with *P*_intercept_ < 0.001; and (**c**) SEF from the right hemisphere (SEFR) with *P*_intercept_ < 0.001. Data are from children (<12 months and 12–36 months) undergoing cardiac surgery with extracorporeal circulation (ECC) under sevolurane anesthesia.

To examine longitudinal changes in BS and SEF over time and between groups, linear mixed-effects models were employed. The primary model included fixed effects of time, age group, and their interaction, with random intercepts for participants to account for repeated measurements. Model assumptions of normality and homoscedasticity of residuals were assessed using Q-Q plots, residuals versus fitted plots, and histograms of residuals. Type III analysis of variance (ANOVA) with Satterthwaite’s approximation for denominator degrees of freedom was applied to confirm the results. Estimated marginal means (EMMs) were computed, and post hoc comparisons of each time point versus baseline were conducted with Bonferroni adjustment. Predicted values and group-level EMMs were plotted to visualize trajectories over time (Figure 1).

**Figure 2:**
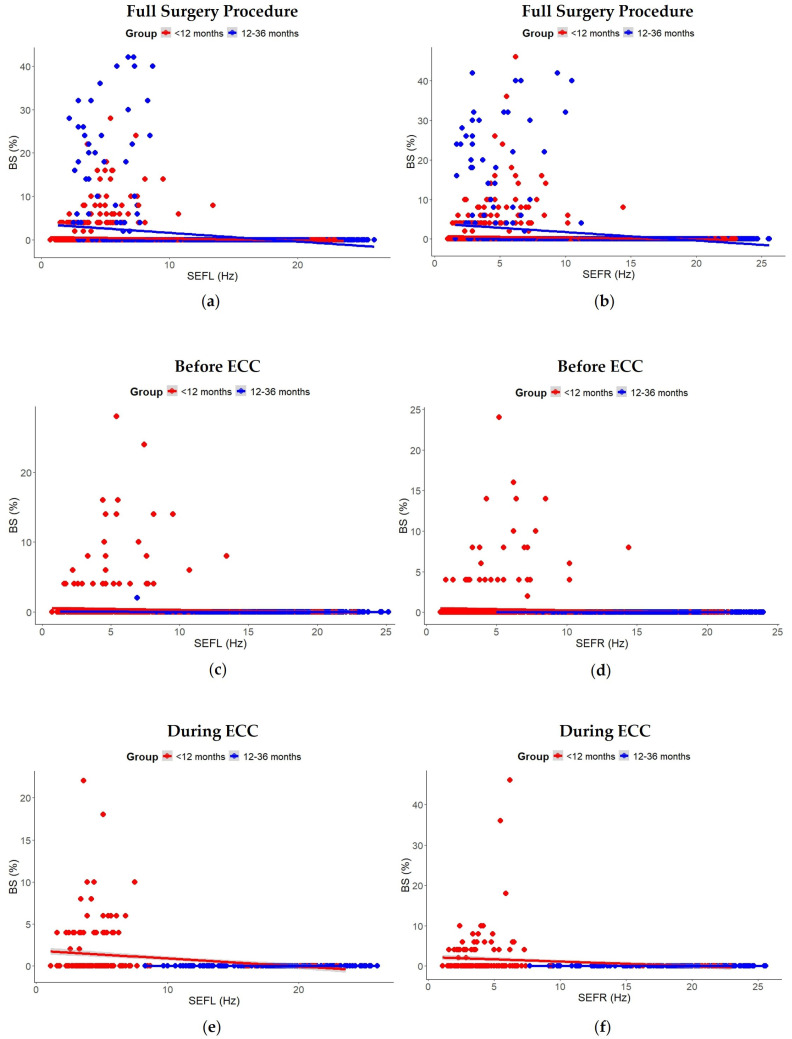
Associations between spectral edge frequency from the left (SEFL) and right (SEFR) hemisphere and frontal cortical burst suppression (BS) by age group. Scatterplots showing associations between BS and (**a**) SEFL (*p* = 0.028) and (**b**) SEFR (*p* = 0.033) during the full surgical procedure; between BS and (**c**) SEFL (*p* > 0.3) and (**d**) SEFR (*p* > 0.3) before extracorporeal circulation (ECC); and between BS and (**e**) SEFL (*p* = 0.25) and (**f**) SEFR (*p* = 0.13) during ECC. Data are from children (<12 months and 12–36 months) undergoing cardiac surgery under sevoflurane anesthesia.

A second linear mixed-effects model was fitted to examine the relationship between BS and SEF from the left (SEFL) and right (SEFR) hemispheres. SEFL and SEFR were included as fixed effects, adjusting for age group, with random interceptions for participants. Model assumptions were assessed using residual plots. Marginal and conditional R^2^ values were calculated to evaluate model fit. Type III ANOVA with Satterthwaite’s approximation was used to confirm results. Predicted values and scatterplots with regression lines were used to visualize the relationship between SEFL/SEFR and BS, stratified by age group (Figure 2).

All analyses were conducted using R (version 4.5.0 “How About a Twenty-Six”, R Foundation for Statistical Computing, Vienna, Austria). Scripts were developed and interpreted with the assistance of Microsoft Copilot, an AI-powered language model based on OpenAI’s GPT-4 architecture. Linear mixed-effects models were fitted using the lmer function from the lme4 package [15], and *p*-values were obtained with the lmerTest package [16]. EMMs and post hoc comparisons were conducted using the emmeans package [17]. Repeated measures correlation plots were generated using the ggplot2 package [18]. All *p*-values were two-tailed and *p* < 0.05 was considered statistically significant.

## 3. Results

### 3.1. Baseline Patient Characteristics

Between January 2020 and June 2024, 12 children aged 6–36 months were included in the study. Patient characteristics for all study participants and in relation to age groups are visualized in Table 1.

During anesthesia, values of EEG/PSi, SpO_2_, HR, MAP, ETCO_2_, and temperature (central and peripheral) were collected. These monitoring parameters are visualized in Table 2 but not included in the subsequent analyses of the current study.

### 3.2. Longitudinal Changes in Burst Suppression and Spectral Edge Frequency

During anesthesia, values of BS and bilateral SEF were collected. These monitoring parameters for all participants and in relation to age group are visualized in Table 3.

#### 3.2.1. Burst Suppression (BS)

Residual diagnostics for the BS model indicated that model assumptions were reasonably met, with no major deviations observed. Most timepoints yielded non-significant *p*-values, suggesting an overall absence of systematic time-dependent variation in BS. However, isolated increases in BS were observed among children in both age groups. Illustrative examples include timepoints at 10 min (β = 5.74, SE = 1.91, *t* = 3.01, *p* = 0.003) and 130 min (β = 5.16, SE = 1.91, *t* = 2.71, *p* = 0.007) for children <12 months, and at 119 min (β = 9.35, SE = 2.64, *t* = 3.54, *p* = 0.0004) and 125 min (β = 9.64, SE = 2.70, *t* = 3.57, *p* < 0.0004) for children aged 12–36 months. Random effects indicated modest between-subject variability (0.28 (SD = 0.532)) relative to within-subject residual variance (6.24 (SD = 2.498)). The Type III ANOVA results showed a similar pattern: no significant main effect of time on BS (*F*(594, 3123.22) = 1.06, *p* < 0.2), no significant main effect of age group (*F*(1, 10.46) = 0.76, *p* = 0.4), and no significant time × age group interaction (*F*(592, 3123.22) = 1.08, *p* = 0.1), indicating that the temporal pattern of change did not differ between groups. None of the Bonferroni-adjusted post hoc comparisons were statistically significant (all *p* = 1.0), indicating that BS did not differ from baseline across time. A scatterplot of predicted BS% over time by group showed stable trends with overlapping group means, supporting the statistical findings (Figure 1a).

#### 3.2.2. Spectral Edge Frequency (SEF)

Residual diagnostics for the SEFL and SEFR models revealed no major deviations from model assumptions. The random intercept variance (SEFL: 24.02; SEFR: 24.48) exceeded the residual variance (SEFL: 14.23; SEFR: 14.20), indicating substantial between-subject variability. Isolated decreases in SEFL were observed among children aged <12 months, notably at 39 min (β = −7.81, SE = 3.08, *t* = −2.53, *p* = 0.01) and 123 min (β = −6.98, SE = 2.88, *t* = −2.11, *p* < 0.04), and in SEFR at 125 min (β = −5.90, SE = 2.88, *t* = −2.05, *p* = 0.04). No decreases in SEFL or SEFR were observed among children aged 12–36 months, with all *p*-values consistently above 0.1. The Type III ANOVA revealed a significant main effect of age group on SEFL (*F*(1, 10.02) = 5.19, *p* < 0.05) and a marginally significant main effect on SEFR (*F*(1, 10.02) = 4.75, *p* = 0.05), indicating that SEF differed between age groups. However, neither the main effect of time (SEFL: *F*(594, 3123.02) = 0.81, *p* = 1.0; SEFR: *F*(594, 3103.02) = 0.81, *p* = 1.0) nor the time × age group interaction (SEFL: *F*(592, 3123.02) = 0.65, *p* = 1.0; SEFR: *F*(590, 3103.02) = 0.61, *p* = 1.0) reached statistical significance. The scatterplots for both SEFL and SEFR over time illustrate the absence of time-dependent variation and highlight the group-level differences. Horizontal lines representing estimated marginal means for each group further emphasized the stability of these metrics across the procedure (Figure 1b,c).

### 3.3. SEF in Relation to BS Throughout the Full Surgical Procedure, Before ECC, and During ECC

#### 3.3.1. Full Surgery Procedure

Mixed-effects models revealed that associations between BS and SEF varied across the surgical phases (Figure 2). Across the full surgical procedure, higher SEFL and SEFR were significantly associated with lower BS (SEFL: β = −0.157, SE = 0.010, *t* = −15.56, *p* < 0.001; SEFR: β = −0.174, SE = 0.011, *t* = −16.13, *p* < 0.001). Age group also had a significant effect, with children aged <12 months consistently exhibiting lower BS compared to those 12–36 months (SEFL: β = −1.166, SE = 0.454, *t* = −2.57, *p* < 0.03; SEFR: β = −1.222, SE = 0.493, *t* = −2.48, *p* = 0.03). Although age group did not significantly affect BS when analyzed over time alone (see Section 3.2.1), it showed a significant effect in models that included SEF as a predictor. This discrepancy likely reflects differences in model structure and highlights the importance of accounting for SEF when assessing age-related differences in cortical suppression. Random intercepts indicated moderate between-subject variability (SEFL: 0.59 (SD = 0.765); SEFR: 0.69 (SD = 0.833)) relative to within-subject residual variance (SEFL: 6.06 (SD = 2.461); SEFR: 6.79 (SD = 2.605), and marginal/conditional R^2^ were 0.117/0.195 for SEFL and 0.126/0.207 for SEFR. The Type III ANOVA confirmed significant effects of both SEF and age group (SEFL: *F*(1, 2569.91) = 242.11, *p* < 0.001; age *F*(1, 9.92) = 6.60, *p* < 0.03; SEFR: *F*(1, 2619.83) = 260.22, *p* < 0.001; age *F*(1, 9.87) = 6.14, *p* = 0.03).

#### 3.3.2. Before ECC

Before ECC, neither SEFL nor SEFR were significantly associated with BS (SEFL: β = −0.005, SE = 0.009, *t* = −0.51, *p* = 0.6; SEFR: β = −0.005, SE = 0.008, *t* = −0.69, *p* > 0.4), and age group had no significant effect (SEFL: β = −0.259, SE = 0.279, *t* = −0.93, *p* > 0.3; SEFR: β = −0.209, SE = 0.215, *t* = −0.98, *p* > 0.3). Random intercepts indicated moderate between-subject variability (SEFL: 0.21 (SD = 0.459); SEFR: 0.12 (SD = 0.351)) relative to within-subject residual variance (SEFL: 1.51 (SD = 1.23); SEFR: 1.05 (SD = 1.023)). Marginal R^2^ values were low (SEFL: 0.012; SEFR: 0.013), whereas conditional R^2^ values were higher (SEFL: 0.133; SEFR: 0.117). The Type III ANOVA confirmed no significant effects of SEF or age group (SEFL: *F*(1, 957.46) = 0.26, *p* = 0.6; age *F*(1, 10.94) = 0.86, *p* > 0.3; SEFR: *F*(1, 760.97) = 0.48, *p* > 0.4; age *F*(1, 11.03) = 0.95, *p* > 0.3).

#### 3.3.3. During ECC

During ECC, mixed-effects models similarly revealed no significant associations between BS and SEF in either hemisphere (SEFL: β = −0.033, SE = 0.040, *t* = −0.84, *p* = 0.4; SEFR: β = −0.043, SE = 0.068, *t* = −0.63, *p* = 0.5). Age group effects were minimal (SEFL: β = −1.070, SE = 0.872, *t* = −1.23, *p* < 0.3; SEFR: β = −2.678, SE = 1.624, *t* = −1.65, *p* = 0.1). Random intercepts indicated substantial between-subject variability (SEFL: 1.71 (SD = 1.308); SEFR: 6.38 (SD = 2.526)) relative to within-subject residual variance (SEFL: 2.75 (SD = 1.658); SEFR: 7.35 (SD = 2.711)), with marginal/conditional R^2^ of 0.094/0.442 for SEFL and 0.138/0.538 for SEFR. The Type III ANOVA confirmed no significant effects of SEF or age group (SEFL: *F*(1, 41.95) = 0.70, *p* = 0.4; age *F*(1, 8.87) = 1.50, *p* < 0.3; SEFR: *F*(1, 67.86) = 0.39, *p* = 0.5; age *F*(1, 9.43) = 2.72, *p* = 0.1).

Scatterplots of SEFL and SEFR versus BS, stratified by age group, illustrated a clear negative relationship across the full procedure, but weak or inconsistent associations before and during ECC, highlighting the phase-dependent nature of SEF–BS dynamics (Figure 2).

## 4. Discussion

In this study, we investigated the relationship between SEF and BS in children undergoing cardiac surgery with ECC. Across the full procedure, higher SEF in both hemispheres was associated with lower BS, reflecting the expected inverse relationship between cortical activity and anesthetic depth. Although age group did not significantly affect BS when analyzed over time alone, the inclusion of SEF in the model revealed a significant age-related difference in BS, suggesting that SEF mediates or interacts with age-related cortical responsiveness to anesthesia. These differences likely reflect maturational changes in neuronal connectivity, synaptic density, and network excitability that influence susceptibility to burst suppression. Previous research supports SEF as a sensitive marker of cortical suppression under general anesthesia. Bong et al. highlighted the clinical utility of EEG monitoring in pediatric anesthesia, emphasizing its potential to optimize anesthesia delivery and enhance patient safety [19]. However, the current study is the first study, to our knowledge, to investigate SEF–BS relationships in children with congenital heart disease undergoing thoracic surgery with ECC under sevoflurane anesthesia. Large multicenter studies in other populations indicate that intraoperative isoelectric events are common in young children and influenced by age, anesthetic technique, and hemodynamic changes [20]. Multichannel EEG studies also demonstrate that brain maturation, particularly thalamocortical circuit development, generates distinct EEG patterns in infants receiving sevoflurane [21]. However, it remains unknown whether intraoperative burst suppression is associated with subsequent neurological sequelae [12]. Our results indicate that the impact of SEF on BS varies with age, with younger children showing a stronger negative correlation, suggesting that the negative SEF–BS relationship may be more pronounced in younger children.

The relationship between SEF and BS was strongly phase dependent. Before and during ECC, neither SEFL nor SEFR significantly predicted BS, and age-related effects were attenuated. These observations suggest that rapid physiological changes, hemodynamic shifts, or the influence of ECC transiently modulate cortical activity, masking the typical SEF–BS relationship observed across the full procedure. Scatterplots illustrated that while SEF and BS were negatively correlated over the entire procedure, associations before and during ECC were weaker or inconsistent. Random intercepts revealed substantial between-subject variability, particularly during ECC, reflecting individual differences in EEG dynamics and anesthetic sensitivity. Marginal R^2^ values indicated that SEF and age explained a modest proportion of BS variance, whereas conditional R^2^ values, incorporating individual variability, substantially improved model fit.

Younger children have immature brains, implying a rapid decline in consciousness under anesthesia [22]. During ECC, surgical stimulation is minimal, and younger children often display lower SEF with a higher risk of BS. This aligns with our findings, underscoring the need for age-adjusted thresholds. The significant interaction between SEF and age suggests that the negative SEF–BS relationship is stronger in infants <12 months, whereas it is less pronounced in older children. Possible explanations include developmental differences in EEG responsiveness, limitations of SEF monitoring equipment in young brains, and the unreliability of autonomic responses as indicators of anesthetic depth. Age-dependent susceptibility to BS underscores the need for age-specific anesthetic strategies and EEG interpretation. Prolonged or repeated exposure to anesthetics in children under three years is associated with potential neurotoxicity, including effects on attention and behavior [22,23]. EEG-guided titration of sevoflurane has been shown to reduce emergence delirium, accelerate recovery, and shorten post-anesthesia care unit stay [24]. The phase-dependent nature of SEF–BS associations indicates that caution is warranted when interpreting SEF during ECC, and complementary measures may be necessary to ensure optimal anesthesia management.

Methodologically, the study employed continuous EEG monitoring and high-resolution data collection, allowing precise characterization of BS dynamics across perioperative phases. For analyses covering the entire surgery procedure, data were down sampled to 10 min intervals, which reduced the data volume and highlighted stable trends, likely enhancing the detection of age-related differences in BS. In contrast, before and during ECC, all measurements were retained at 20 s resolution, preserving rapid fluctuations in EEG signals. The use of linear mixed-effects models accounted for repeated measures and inter-individual variability, providing robust estimates of SEF–BS relationships across phases and age groups while demonstrating that aggregation level can influence the apparent effects of age and SEF.

Clinically, these results highlight the potential utility of SEF monitoring as a non-invasive tool to assess anesthetic depth in young children. The phase-dependent nature of SEF–BS associations suggests that caution is warranted when interpreting SEF in the context of ECC, and that complementary measures may be needed to ensure optimal anesthesia management. Furthermore, the observed age differences in BS susceptibility could inform age-specific anesthetic strategies and EEG interpretation, particularly in children younger than 12 months.

A strength of the current study is the use of continuous, high-resolution EEG monitoring combined with advanced statistical modeling, which enabled a detailed characterization of SEF–BS dynamics across perioperative phases. The main limitation is the small sample size (*n* = 12), with only six children <12 months and six children between 12 and 36 months, which limits the generalizability of the findings. Another limitation is that EEG recordings were not obtained while the children were awake and prior to the induction of anesthesia, as we aimed to avoid unnecessary stress for the patients in the pre-induction phase.

Overall, our findings contribute to a better understanding of how cortical activity and anesthetic depth interact in young children undergoing complex cardiac surgery. They emphasize the importance of considering both age and perioperative phase when monitoring EEG indices, and they provide a foundation for further research on individualized anesthetic management using neurophysiological biomarkers. The results highlight the need for precision medicine in pediatric anesthesia, emphasizing the importance of individualized care, proactive identification of potential side effects, and risks and the necessity for accurate and tailored monitoring [25].

## 5. Conclusions

The current single-center observational study suggests that spectral edge frequency (SEF) in the low-frequency range may serve as a valuable, age-sensitive neurophysiological marker for detecting burst suppression (BS) in young children undergoing cardiac surgery with sevoflurane anesthesia. The findings demonstrate that younger children exhibit more BS at comparable SEF values than older children. These results highlight the need for age-adjusted anesthesia monitoring strategies, as identical SEF thresholds do not reflect equivalent levels of cortical suppression across pediatric age groups.

## Figures and Tables

**Table 1:** Baseline patient characteristics for all and in relation to age groups.

	All	<12 Months	12–36 Months
	*n* = 12	*n* = 6	*n* = 6
	*n* (%) or mean (SD)	*n* (%) or mean (SD)	*n* (%) or mean (SD)
Gender:			
Female	9 (75.0)	3 (50.0)	6 (100.0)
Male	3 (25.0)	3 (50.0)	0 (0.0)
Height, cm	75.4 (13.40)	64.8 (5.57)	89.5 (1.87)
Weight, kg	8.7 (3.08)	6.3 (1.43)	11.8 (0.85)
BSA, m^2^	0.41 (0.12)	0.32 (0.05)	0.54 (0.02)
Diagnosis:			
ASD	5 (41.7)	1 (16.7)	4 (66.7)
VSD	3 (25.0)	1 (16.7)	2 (33.3)
ASD, VSD	3 (25.0)	3 (50.0)	0 (0.0)
ASD, VSD, PDA	1 (8.3)	1 (16.7)	0 (0.0)

Abbreviations: ASD, atrial septal defect; BSA, body surface area; PDA, patent ductus arteriosus; VSD, ventricular septal defect.

**Table 2:** Intraoperative monitoring parameters.

	All	<12 Months	12–36 Months	
	*n* = 12	*n* = 6	*n* = 6	
	Mean (SD)	Mean (SD)	Mean (SD)	*p*-value *
EEG/PSi	35.8 (17.16)	33.1 (19.30)	39.1 (13.47)	0.3
HR, bpm	110.3 (19.21)	116.6 (19.87)	104.2 (16.38)	0.04
SpO_2_, %	95.88 (3.103)	95.50 (3.148)	96.65 (2.847)	0.1
ETCO_2_, kPa	5.14 (1.379)	4.73 (1.294)	5.46 (1.356)	0.2
MAP, mmHg	48.8 (11.09)	46.8 (11.87)	51.2 (9.63)	0.04
Temperature, °C:				
Esophageal	35.7 (0.91)	35.7 (1.07)	35.7 (0.55)	0.9
Rectal	35.7 (0.97)	35.4 (1.10)	36.1 (0.53)	0.1
Skin	31.9 (3.00)	30.8 (3.13)	33.8 (1.46)	0.09

Abbreviations: EEG, electroencephalogram; ETCO_2_, end-tidal carbon dioxide; HR, heart rate; MAP, mean arterial pressure; PSi, patient state index; SpO_2_, oxygen saturation. * *p*-value corresponding to the age group effect in the Type III ANOVA model.

**Table 3:** Intraoperative values of burst suppression and spectral edge frequency.

	All	<12 Months	12–36 Months	
	*n* = 12	*n* = 6	*n* = 6	
	Mean (SD)	Mean (SD)	Mean (SD)	*p*-value *
Full surgery procedure:				
Time, minutes	162.4 (38.37)	172.1 (34.54)	152.7 (42.67)	0.4
BS, %	3.8 (15.70)	5.3 (19.55)	2.1 (9.04)	0.4
SEFL, Hz	13.2 (6.69)	10.3 (6.90)	16.5 (4.63)	<0.05
SEFR, HZ	13.2 (6.65)	10.4 (6.91)	16.4 (4.65)	0.05
Before ECC:				
Time, minutes	72.4 (32.55)	68.1 (31.22)	76.7 (36.21)	0.7
BS, %	0.4 (4.63)	0.3 (1.73)	0.5 (6.34)	0.6
SEFL, Hz	13.5 (5.96)	10.0 (6.50)	16.6 (3.03)	0.03
SEFR, HZ	13.5 (5.81)	10.1 (6.37)	16.5 (2.88)	0.04
During ECC:				
Time, minutes	20.7 (18.37)	30.8 (21.41)	11.0 (6.95)	0.06
BS, %	19.5 (34.67)	27.0 (38.25)	0.0 (0.0)	0.07
SEFL, Hz	14.9 (7.67)	12.0 (7.72)	19.8 (4.45)	0.04
SEFR, HZ	14.6 (7.90)	11.8 (8.02)	19.6 (4.55)	0.04

Abbreviations: BS, burst suppression; ECC, extracorporeal circulation; SEFL, spectral edge frequency left; SEFR, spectral edge frequency right. * *p*-value corresponding to the age group effect in the Type III ANOVA model.

## Data Availability

The data presented in this study are available on request from the corresponding author due to ethical reasons.

## Abbreviations

The following abbreviations are used in this manuscript:

ANOVA	Analysis of variance
ASA	American society of anesthesiologists
ASD	Atrial septal defect
BS	Burst suppression
BSA	Body surface area
CPB	Cardiopulmonary bypass
CHD	Congenital heart disease
˚C	Degrees Celsius
ECC	Extracorporeal circulation
EEG	Electroencephalography
EMM	Estimated marginal mean
ETCO_2_	End-tidal carbon dioxide
HR	Heart rate
Hz	Hertz
kPa	Kilopascal
MAC	Minimum alveolar concentration
PDA	Patent ductus arteriosus
PSi	Patient state index
SD	Standard deviation
SE	Standard error
SEF	Spectral edge frequency
VSD	Ventricular septal defect
